# Using remotely sensed data to identify areas at risk for hantavirus pulmonary syndrome.

**DOI:** 10.3201/eid0603.000303

**Published:** 2000

**Authors:** G E Glass, J E Cheek, J A Patz, T M Shields, T J Doyle, D A Thoroughman, D K Hunt, R E Enscore, K L Gage, C Irland, C J Peters, R Bryan

**Affiliations:** The Johns Hopkins School of Hygiene and Public Health, Baltimore, Maryland 21205, USA.

## Abstract

The 1993 U.S. hantavirus pulmonary syndrome (HPS) outbreak was attributed to environmental conditions and increased rodent populations caused by unusual weather in 1991- 92. In a case-control study to test this hypothesis, we estimated precipitation at 28 HPS and 170 control sites during the springs of 1992 and 1993 and compared it with precipitation during the previous 6 years by using rainfall patterns at 196 weather stations. We also used elevation data and Landsat Thematic Mapper satellite imagery collected the year before the outbreak to estimate HPS risk by logistic regression analysis. Rainfall at case sites was not higher during 1992-93 than in previous years. However, elevation, as well as satellite data, showed association between environmental conditions and HPS risk the following year. Repeated analysis using satellite imagery from 1995 showed substantial decrease in medium- to high-risk areas. Only one case of HPS was identified in 1996.


### Using Remotely Sensed Data To Identify Areas at Risk For Hantavirus Pulmonary Syndrome

Gregory E. Glass,* James E. Cheek,† Jonathan A. Patz,* Timothy M. Shields,* Timothy J. Doyle,‡ Douglas A. Thoroughman,† Darcy K. Hunt,† Russell E. Enscore,§ Kenneth L. Gage,§ Charles Irland,† C. J. Peters,¶ and Ralph Bryan§
*The Johns Hopkins School of Hygiene and Public Health, Baltimore, Maryland, USA; †Indian Health Service, Albuquerque, New Mexico, USA; ‡Centers for Disease Control and Prevention, Albuquerque, New Mexico, USA; §Centers for Disease Control and Prevention, Ft. Collins, Colorado, USA; and ¶Centers for Disease Control and Prevention, Atlanta, Georgia, USA

The 1993 U.S. hantavirus pulmonary syndrome (HPS) outbreak was attributed to environmental conditions and increased rodent populations caused by unusual weather in 1991-92. In a case-control study to test this hypothesis, we estimated precipitation at 28 HPS and 170 control sites during the springs of 1992 and 1993 and compared it with precipitation during the previous 6 years by using rainfall patterns at 196 weather stations. We also used elevation data and Landsat Thematic Mapper satellite imagery collected the year before the outbreak to estimate HPS risk by logistic regression analysis. Rainfall at case sites was not higher during 1992-93 than in previous years. However, elevation, as well as satellite data, showed association between environmental conditions and HPS risk the following year. Repeated analysis using satellite imagery from 1995 showed substantial decrease in medium- to high-risk areas. Only one case of HPS was identified in 1996.

In 1993, a disease characterized by acute respiratory distress with a high death rate (>50%) among previously healthy persons was identified in the southwestern United States. This disease, hantavirus pulmonary syndrome (HPS), was traced to infection with an unrecognized, directly transmissible virus—Sin Nombre virus (SNV) (Bunyaviridae; Hantavirus) (1). The virus was maintained and transmitted primarily within populations of a common native rodent, the deer mouse (***Peromyscus maniculatus***) (2), and transmission to humans occurred through contact with secretions and excretions of infected mice (3).

It has been hypothesized that the El Niño Southern Oscillation (ENSO) of 1991-92 was the major climatic factor producing environmental conditions leading to the outbreak of HPS in 1993. Unseasonable rains in 1991 and 1992 during the usually dry spring and summer and the mild winter of 1992 are thought to have created favorable conditions for an increase in local rodent populations (4,5).

This hypothesis is based primarily on the following observations: 1) ENSO tends to influence the timing and abundance of precipitation in the southwestern United States. 2) Some ***Peromyscus*** populations increased dramatically in areas where precipitation was above average but remained near normal levels where precipitation did not increase—this observation is based on comparison of data from only two study areas: the University of New Mexico's Sevilleta Long-Term Ecological Research station (90 km south of Albuquerque, NM), where precipitation was 2 to 3 times the previous

Address for correspondence: Gregory E. Gurri Glass, Molecular Microbiology & Immunology, The Johns Hopkins School of Public Health, 615 N. Wolfe Street, Baltimore, MD 21205, USA; fax: 410-955-0105; e-mail: gurriglass@msn.com.

20-year average, and Moab, Utah, where precipitation was at or below normal in the summer of 1992. Before and during the HPS outbreak, populations of ***P. maniculatus*** did not increase in Moab, while at sites on the Long-Term Ecological Research station they were 10 to 15 times higher than normal. Moreover, both Moab and the research station were 200 km to 300 km from the epicenter of the 1993 HPS outbreak, making comparison with conditions where disease occurred uncertain. 3) 1993 case studies found that rodent abundance varied dramatically over short distances. Rodent populations were higher at HPS households than at neighboring households without disease or randomly selected households at least 25 km away (6); however, these one-time case studies provide little information on the responses of rodent populations to changes in environmental conditions. Although associating weather with HPS outbreaks is consistent with these observations, supporting data are limited. This reflects the situation for many emerging diseases, as active surveillance for unidentified diseases is rare.

Current attempts to understand the factors leading to HPS outbreaks focus on detailing the chain of events from weather, through changes in vegetation, to virus maintenance and transmission within rodent populations, culminating in changes in human disease risk (trophic cascade hypothesis) (4,5,7). An impediment to this approach, however, is the focal nature of SNV within local populations of ***P. maniculatus***. Among local rodent populations the rates of infection vary, and some populations appear uninfected (8). The reason for this is uncertain but may be related to the stochastic loss of the horizontally transmitted viruses within local populations of the reservoir (9). Alternatively, the dynamics of local populations may be such that SNV cannot be maintained at very low population numbers. Under either circumstance, responses of local populations to environmental fluctuations could substantially alter human risk. Given the sporadic nature of HPS outbreaks, ongoing, longitudinal monitoring of rodent populations in the vicinity of subsequent cases is unlikely (6). Consequently, inferring human disease risk from rodent-SNV population dynamics will again require extrapolating from studies in regions other than the site of the outbreak.

In this article, we examine the relationship of the environment to HPS risk by using locations of HPS cases as sites where people were associated with infected rodents. This approach avoids the immediate need to establish the conditions that lead some reservoir populations to be uninfected by SNV. We compare the environmental characteristics of sites where people were infected with those at sites where people were not infected. Differences in environmental conditions could indicate factors that influence either the abundance of rodents or the occurrence of virus, creating testable hypotheses of environmental conditions that influence SNV infection patterns in reservoir populations. As a partial test of our method, the analysis was repeated when cases of HPS were uncommon. Under these conditions, the identified environmental factors should indicate low levels of disease risk.

Two sources of information were used as measures of the local environmental conditions preceding the HPS outbreak: 1) Monthly patterns of precipitation from March to June (generated from archived weather station data in the region to estimate local precipitation patterns at both case and control sites) and 2) satellite imagery (obtained before the HPS outbreak and used as a measure of variable, local environmental conditions and HPS risk evaluated by epidemiologic analysis).

### Methods

### Study Population and Region

The epidemiologic analysis was performed as a case-control study. Twenty-eight (93.3%) of 30 sites with confirmed cases of HPS identified in the region between November 1992 (identified retrospectively) and November 1994 were selected. Inclusion criteria were based on clinical disease consistent with the Centers for Disease Control and Prevention case definition that was confirmed by serologic, nucleic acid, or immunohistochemical tests (1). One case was excluded because the likely site of exposure could not be established, and a second case because we could not confirm that the proper geographic location of the site was recorded during data collection.

Sites of exposure were established previously by investigation of each case-patient's activities and, for most fatal cases, demonstration of sequence homology of polymerase chain reaction (PCR)-amplified regions of SNV nucleic acids obtained from case-patients and rodents collected at the imputed sites of exposure (1,2). Sites of exposure for the HPS cases were at or in the immediate vicinity of households, and there usually was a history of activities that could have generated exposure to contaminated aerosols (1).

To control for issues related to access to care and socioeconomic conditions, controls were selected randomly from all households that used the same health clinics as the HPS patients during the same period as the HPS patients. Controls were randomly selected among persons without HPS. A total of 170 persons with different residential addresses were identified from a listing of visits to all the clinics during the time of the HPS outbreak. This represented an approximately 2% random sample of addresses of the patient population. A previous study showed that subclinical infection with SNV was not observed among controls (10). Geographic locations of case and control sites were established by using global positioning system (GPS) receivers to record latitude and longitude at each site.

The study area of 105,200 km$^2$ was located in the southwestern United States, incorporating the region of the original HPS outbreak (1,2). Epidemiologic surveillance was part of the HPS outbreak investigation.

### Environmental Characterization of Sites

Monthly precipitation data recorded at 196 weather stations throughout the region from 1986 to 1993 were from the U.S. National Oceanic and Atmospheric Administration's National Climatic Data Center. Spring precipitation at the weather stations was calculated by aggregating monthly precipitation for March through June. Spring precipitation at individual case and control sites was estimated by interpolation among the nearest weather stations to each case or control site. Interpolation procedures used the eight nearest weather stations to estimate precipitation by applying trend surface algorithms from the GIS software (IDRISI, 11). Spring precipitation during 1992 to 1993 at each site was compared with spring precipitation during previous years by paired differences tests, with correction for multiple tests. The null hypothesis for each case or control site was that there was no increase in precipitation during the spring of 1992 to 1993 compared with the previous years. We also used satellite imagery to develop detailed characterization of local environmental conditions. We selected three archived Landsat Thematic Mapper (TM) images originally recorded in mid-June 1992 for analysis. TM records digital numbers (DN) from reflected light in six bands, three in visible and three in infrared (IR) portions of the electromagnetic (EM) spectrum. (An additional band of thermal IR energy also is recorded but was not used.)

The TM images were merged to form the study area of 105,200 km.$^2$ Nominal pixel resolution was 30 m. Images were geometrically and radiometrically corrected by the U.S. Geological Survey (USGS) Earth Resources Observation Systems (EROS) Data Center. The images were imported into a raster-based geographic information system (GIS) for geographic registration by using control points obtained from USGS quadrangle maps (11). The images were resampled by a bilinear resampling procedure with a quadratic mapping function (11). Corrections for atmospheric scattering of bands one to three were performed by the method of Chavez (12). Latitude and longitude positions of case and control households were imported as a data layer in IDRISI GIS. Additional environmental variables incorporated in GIS included elevation, slope, and aspect of the case and control sites. Elevation was derived from the USGS digital elevation models (1:250,000 scale), while slope and aspect were generated from these data by using software in GIS.

Three satellite images from mid-June 1995 were selected to further validate the analysis of the 1992 imagery. There was no ENSO from 1994 to 1996, and we predicted that any relationship between the satellite imagery in 1992 and the case and control sites would indicate reduced risk in 1995. DNs for selected locations on the 1992 and 1995 images were compared to determine any significant changes in sensor calibration.

### Epidemiologic Analysis of HPS by Satellite Imagery

The spatial distribution of HPS sites (cases) was compared with that of control sites to determine if cases were spatially aggregated within the study region (13). Then, the relationship between HPS and environmental factors measured by TM imagery was examined. Because the analysis used three tiled images, the strategy for model development involved model fitting by using a portion of the study region, followed by external validation (14). A sample of HPS sites and control sites was selected for analysis, by using logistic regression to examine the relationship between the odds of a site being an HPS site (outcome variable) and the DN in each TM band, elevation, slope, and aspect (predictor variables). The analysis was then repeated by using the remaining HPS and control sites to validate the model. Identifying the same model in the two analyses would indicate that the HPS risk model was robust for that period.

The initial model used a test area of 12,279 $km^2$ from the east-central region of the study area. This area included 14 case and 36 control sites and was entirely within one TM image. The validation analysis used the remaining sites (14 case and 134 control sites) and incorporated all or parts of the three TM images covering 92,921 $km.^2$ The average DN for each of the six TM bands used in the analysis was calculated with a 3 x 3 pixel filter centered on the location for each case or control site. This sampled a local region of approximately 8,100 $m^2$ around each case or control site.

We used logistic regression analysis to identify the best combination of TM bands and environmental variables associated with HPS status (14). Elevation was dichotomized at the median elevation for case and control sites, combined. Inclusion of remotely sensed variables was based on the statistical significance of their coefficients in the model. Elevation was retained in all models because of its observed association with ***P. maniculatus*** abundance (8). The logistic model was evaluated with the Deviance and the Hosmer-Lemeshow goodness of fit statistics (C) for deciles of risk. To examine the accuracy of the model predictions, we evaluated the sensitivity and specificity by creating a Receiver Operator Characteristic function (15). The function compares the true-positive rate (sensitivity) against the false-positive rate (1 – specificity) of a model by using various predicted values as thresholds identifying case and control sites. In addition to examining spring precipitation, we evaluated the trophic cascade hypothesis by examining the relationship between HPS risk and vegetation growth before the HPS outbreak (5), regardless of habitat type. We used data from the near infrared (band 4) and red (band 3) portions of the spectrum to generate a normalized difference vegetation index of the region. The index is a standard algorithm used as a measure of vegetative growth. We compared the index and the TM bands identified in the initial epidemiologic analysis for estimating HPS risk by comparing the Receiver Operator Characteristic's generated by each analysis.

### Results

Cases and controls occupied the same general, geographic area with the greatest difference of most extreme sites of 20 km in the north-south and 13 km in east-west directions. Despite the broad geographic overlap, cases of HPS were not randomly distributed within the area. HPS cases were spatially clustered (Table 1). Despite this clustering, HPS sites were widely separated geographically. The average distance between case sites was 50.3 km (SD = 23.8 km), and the nearest neighboring sites (k = 1; Table 1) were not themselves likely to be case sites.

Table 1. Spatial clustering of households with hantavirus pulmonary syndrome

| k | T[k] | z | P |
|---|---|---|---|
| 1 | 6 | 0.91 | 0.181 |
| 2 | 14 | 1.88 | 0.030 |
| 3 | 24 | 3.05 | 0.001 |
| 4 | 33 | 3.71 | <0.001 |
| 5 | 40 | 3.87 | <0.001 |
| 6 | 50 | 4.57 | <0.001 |
| 7 | 64 | 5.85 | <0.001 |
| 8 | 75 | 6.50 | <0.001 |
| 9 | 79 | 6.14 | <0.001 |
| 10 | 86 | 6.21 | <0.001 |

Cuzick and Edwards' test (13), T[k], its z score under the null hypothesis and the p value using Simes correction for multiple tests (16). Results show significant spatial clustering among case households for the second through tenth nearest neighbors (k= 2,...,10).

Spring precipitation patterns showed substantial interannual variation at case and control sites (Figure 1). From 1986 through 1993, precipitation was 4.5 mm (1989) to 110 mm (1992). Spring precipitation decreased markedly between 1992 and 1993 (Figure 1). Overall, precipitation at control sites tended to be lower (65 mm) than at case sites (72 mm) during the spring each year, but there was broad overlap among sites, and yearly variation at control sites tracked that of the case sites (Figure 1). None of the case sites had higher precipitation during 1992 to 1993 than during the preceding 6 years ($p > 0.05$).

There was a significant relationship between local environmental conditions and HPS risk as measured by the statistical association between

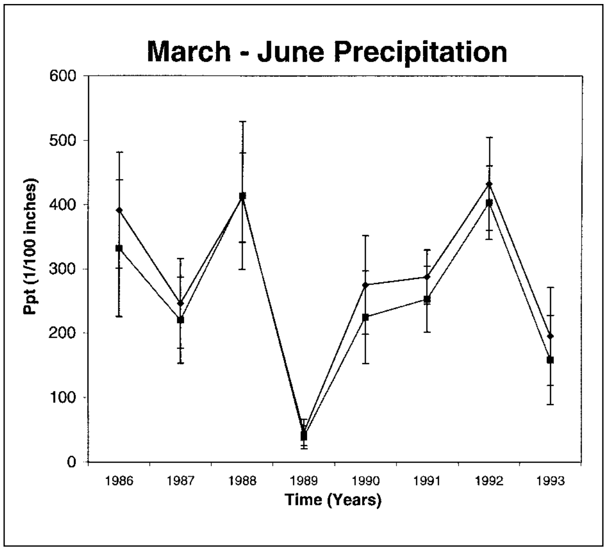

Figure 1. March–June precipitation patterns at case sites (solid symbols) and control sites (open symbols) from 1986 through 1993. Vertical bars are 1 standard deviation in precipitation values.

the DN recorded by the satellite in 1992 and HPS risk the following year. The logistic regression analysis developed for the training area fit the observed data well (Deviance = 45.45; p = 0.49, df = 46; Hosmer Lemeshow C = 6.62; p = 0.58, df = 8), and none of the sites were obvious outliers. Higher level interactions between the independent variables did not change the results. Three of the six bands from the TM images (1, 5, and 7) were associated with the odds of HPS (Table 2). Sites above the median elevation (2,094 m) were marginally associated with risk in the training area, but elevation was retained because of the relatively small sample sizes used to estimate the parameters, as well as the biologic rationale outlined elsewhere (8).

High DN values in the blue (band 1) and mid-infrared (band 7) portions of the EM spectrum were associated with decreased risk for HPS, while high values in the mid-infrared (band 5) portion of the spectrum were a risk factor for HPS (Table 2). The DN values were approximately 58 to 233 units. Each unit change in the average DN around sites altered the odds of HPS risk by 6% (band 1), 15% (band 7). Sites above 2,094 m in the test area were >4 times as likely to be HPS sites as sites below 2,094 m. Slope and aspect at the sites were not associated with risk.

The Receiver Operator Characteristic graph of sensitivity and specificity of the predictor function showed that at least 95% of the case sites were correctly identified until the proportion of control sites correctly identified exceeded 56% (20 of 36 control sites) (Figure 2). The same predictor variables from the TM imagery were identified when the analysis was repeated for the validation area. The coefficients of the validation analysis did not differ significantly from those for the training area (Table 2). This model also fit the data well (Deviance = 74.49; p = 1.00, df = 144; Hosmer Lemeshow C = 6.78; p =0.56, df = 8).

Because the logistic models did not differ between the training and validation areas, all the

Table 2. Logistic regression analysis of LANDSAT Thematic Mapper data and elevation

| | Training area | | | Validation area | | | |
|---|---|---|---|---|---|---|---|
| Predictor | Odds ratio | 95% CI | P | Odds ratio | 95% CI | P | t |
| Band 1 | 0.94 | (0.89, 0.99) | 0.027 | 0.95 | (0.90, 0.99) | 0.019 | ns |
| Band 5 | 1.14 | (1.03, 1.26) | 0.013 | 1.08 | (1.01, 1.17) | 0.034 | ns |
| Band 7 | 0.85 | (0.73, 0.99) | 0.039 | 0.90 | (0.80, 1.00) | 0.046 | ns |
| Elevation | 4.63 | (0.88, 24.29) | 0.070 | 2.56 | (0.77, 8.43) | 0.123 | ns |

| | Overall | | | Controls | | Cases | |
|---|---|---|---|---|---|---|---|
| Predictor | Odds ratio | 95% CI | p | Mean | Range | Mean | (Range) |
| Band 1 | 0.96 | (0.93, 0.99) | 0.012 | 111.9 | (70.6, 164.0) | 97.8 | (72.0, 134.3) |
| Band 5 | 1.07 | (1.01, 1.13) | 0.013 | 170.6 | (110.7, 253.3) | 160.7 | (110.2,226.2) |
| Band 7 | 0.91 | (0.84, 0.99) | 0.015 | 102.6 | (60.6, 163.3) | 93.1 | (58.2, 140.4) |
| Elevation | 4.60 | (1.96, 10.79) | 0.005 | 1935 | (1465, 2500) | 2081 | (1825, 2341) |

Odds ratios, 95% confidence intervals, and statistical significance of HPS predictor variables. Odds ratios of the Thematic Mapper bands indicate the change in risk for each unit change in digital number recorded by the satellite sensor. Elevation was divided as either above or below 2,094 m for the analysis. There was no difference between the Training and Validation areas, as tested by t-test (t). The Controls and Cases columns show the mean and range of values at the control and case sites, respectively.
ns=not shown.

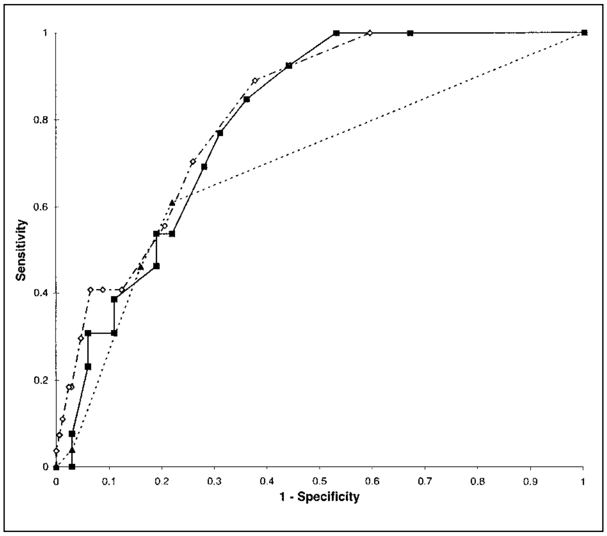

Figure 2. Receiver Operator Characteristic (ROC) function of the logistic model for HPS risk in the training area (closed squares) and overall for 1992 (open diamonds) as the threshold for predicted case households was varied from p = 0.00 - 0.85 in 0.05 increments. The ROC for normalized difference vegetation index model (triangles) had p values of 0.00 to 0.40 and multiple points occurred together. The early rapid loss of sensitivity for the NDVI model was the result of poor model specification.

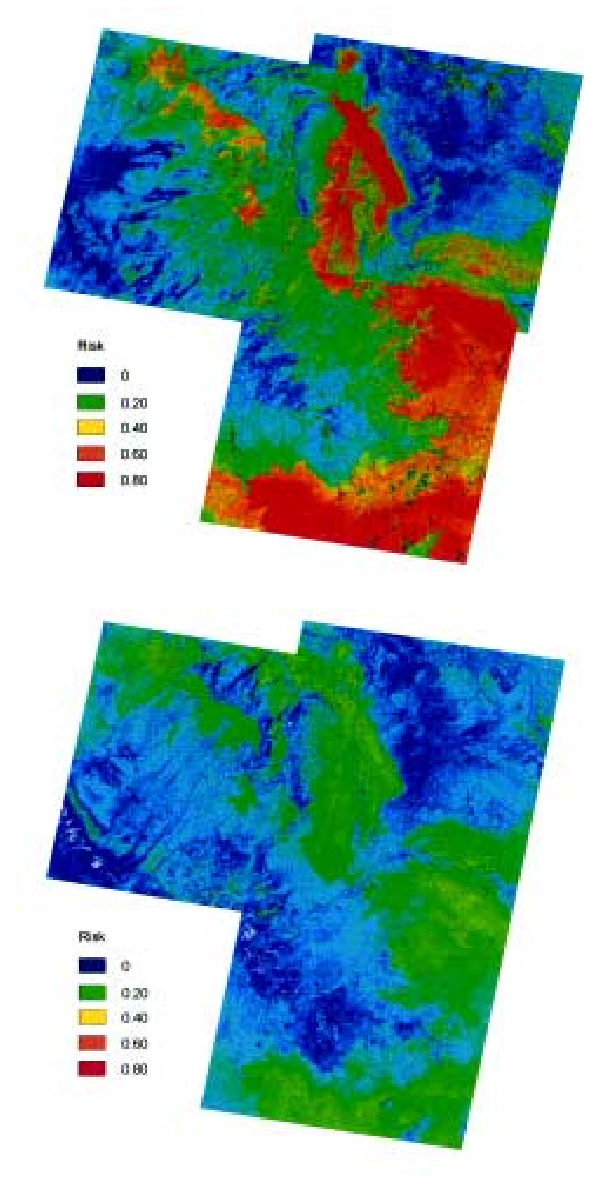

Figure 3. Comparison of predicted HPS risk for 1993 (top) and 1996 (bottom) by satellite imagery taken in 1992 and 1995, respectively, in the study area. Low-risk areas are in dark blue and high-risk areas are in red and yellow. There was a significant reduction in predicted high-risk areas in 1996 compared with 1993.

sites were combined to give an overall model (Table 2). The overall Receiver Operator Characteristic (Figure 2) had a sensitivity and specificity similar to those of the test area (95% sensitivity, 62% specificity). This threshold corresponded to a predicted value for HPS of approximately 0.10. Thus, using a predicted log odds ratio of at least 0.10 as a threshold for increased risk included 95% of the case sites and excluded 62% of the control sites.

The logistic function was applied to each pixel in the study area to produce a map of predicted risk (Figure 3) (17). The analysis was repeated by using satellite images from June 1995 to predict HPS risk for 1996 (Figure 3). There was a near elimination of predicted high-risk areas in the 1995 imagery and a broad expansion of low-risk areas compared with the 1992 images. The single case of HPS reported from the region in 1996 occurred at a site with a predicted risk of 0.16 (i.e., above the HPS threshold).

Areas of high vegetation growth in 1992, as measured by the normalized difference vegetation index (Figure 4) incorporated only a portion of the HPS high-risk areas (Figure 3). We evaluated vegetation growth, as measured by the index, as a predictor of HPS risk by modeling the case-control data, using the index and elevation as predictor variables. The vegetation growth model that best fit the observed data included an

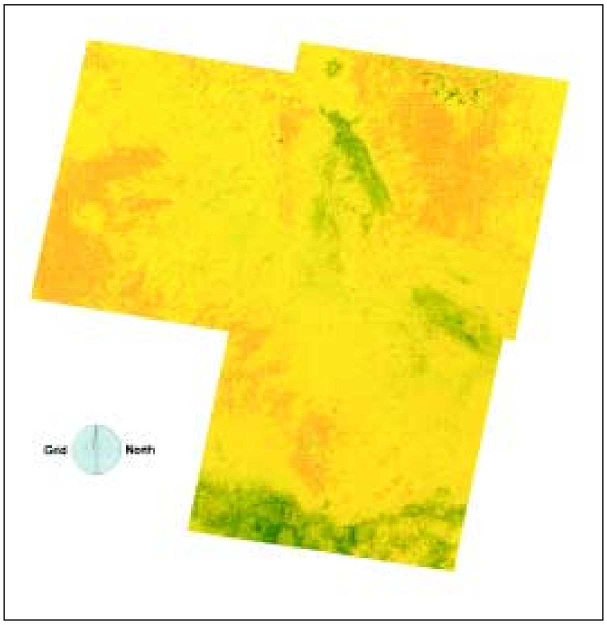

Figure 4. The normalized difference vegetation index (NDVI) scores of the study area by Thematic Mapping bands 3 and 4. Vegetation growth increased from brown through yellow to green. There was a substantial portion of high-risk area (especially the eastern portion of the image) where the NDVI image pixels did not obviously correspond to high-risk areas (see Figure 3 for comparison).

exponential transformation of the normalized difference vegetation index and sites above the median elevation. This index model accounted for a significant part of the variation in the HPS risk (deviance = 147.62; p = 0.99, df = 196) but did not accurately model the odds of HPS. In this analysis 11 (39.3%) of 28 case sites had standardized residuals exceeding three standard deviations, suggesting a poor fit to the data—an interpretation supported by the Hosmer Lemeshow statistic (C = 20.09; p = 0.01, df = 8), which indicated that the form of risk model fit the data poorly. The receiver operator characteristic of the vegetation index analysis (Figure 2) also lost sensitivity more rapidly than the analysis using TM bands 1, 5, and 7, especially over the range of values near the threshold of increasing HPS risk.

### Conclusions

Satellite imagery, combined with epidemiologic surveillance, retrospectively identified areas at high risk for HPS associated with ***Peromyscus*** populations over broad geographic regions during the 1993 outbreak. TM data identified environmental conditions near HPS sites that were measurably different from conditions in rural, populated sites where disease did not occur for nearly 1 year before the outbreak. These environmental conditions varied with the presence of ENSO (Figure 3). The geographic extent and general level of predicted HPS risk were higher during ENSO, supporting the view that El Niño may increase the likelihood of HPS outbreaks. The hypothesized pathway between ENSO, increased spring precipitation leading to increased vegetation growth, and subsequent HPS risk, however, was not strongly supported by the data. Possible reasons for this lack of support are discussed below.

In this study, we used a retrospective epidemiologic approach to risk assessment (15). Therefore, odds ratios of the environmental characteristics were used to estimate the population's relative risk for HPS. This approach is valid when the cases used in the study are representative of all cases, the controls are representative of the general population, and the disease is relatively rare. Under these conditions, odds ratios approximate relative risk (15).

HPS is rare–fewer than 1,000 cases have been identified in North America, although most occurred in the southwestern region of the United States. In this study, the cases included nearly all (28 of 30) sites in the region where HPS occurred during the outbreak. Although bias induced from this factor is unlikely, we cannot be certain that environmental factors identified with outbreaks of HPS are similar to those with the sporadic, single cases of HPS reported each year. However, the accurate identification of the site where the single case of HPS was observed in 1996 suggests that the classification may also identify risk characteristics for this group. Issues related to personal privacy make accessing these data difficult, however, because geographic locations of residences, for example, are considered personal identifiers.

The selection of controls focused on a population from the same socioeconomic and geographic region as the HPS cases. Although HPS cases were clustered, the maximal geographic extent of both cases and controls was similar, suggesting that the enrollment procedure adequately fulfilled this goal. Random selection of controls also was intended to control for access to care in a region where travel may be difficult. Restricting controls to those using the same health-care facilities again raises the issue of the applicability of the results to areas with different socioeconomic and cultural conditions and probably excluded much of the population within urban areas of the study site.

These are relatively minor potential problems. HPS cases are rare in urban areas because the primary reservoir species in North America rarely occur within urban settings. Moreover, surveys of rural housing show that infestations by ***Peromyscus*** are nearly ubiquitous in the absence of focused rodent exclusion methods (6,18).

Absence of a significant difference in spring precipitation at case sites during 1992-93 and the previous 6 years (Figure 1) may reflect either the absence of an effect (contradicting the trophic cascade hypothesis) or practical problems with estimating precipitation and incorporating conditions associated with past HPS outbreaks. Although nearly 200 weather stations were used in estimating spring precipitation at case and control sites, this still represents a relatively sparse network of sampling locations; therefore, localized precipitation could have been at too fine a spatial scale to detect. However, when we estimated precipitation at weather stations by using the surrounding stations and comparing the results with the observed precipitation, we found no difference between observed and predicted results in 1992-93. A more likely possibility is that the relatively short time series of precipitation data used makes demonstrating a statistical effect difficult. Additionally, if ENSO is a triggering event, outbreaks of HPS must have occurred in the past. Therefore, previous ENSO events may "contaminate" comparisons with past precipitation data because they include unrecognized HPS outbreaks.

The trophic cascade hypothesis predicts that ENSO leads to increased precipitation that affects vegetation growth, subsequently influencing HPS risk. The association between HPS risk and vegetation growth, as measured by the normalized difference vegetation index, was inconsistent. Areas with a high index (Figure 4) did correspond to areas at highest risk for HPS (Figure 3) in 1992. Similarly, areas at low risk were generally those with low normalized difference vegetation indexes. However, broad regions of moderate- to high-risk areas did not relate to the vegetation index, and the logistic regression model did not perform well (Figure 2).

The failure of the normalized difference vegetation index to predict HPS risk may indicate that the ecologic connections hypothesized by the trophic cascade hypothesis are complex and modulated by intervening ecologic variables. Alternatively, the normalized difference vegetation index, which is the normalized difference of DN in red and near-infrared portion of the EM spectrum, may have difficulty accurately characterizing vegetation growth in semi-arid areas that contain a complex mixture of vegetation and bare ground (19). Further, detailed studies incorporating "ground truthing" to establish the relationship between local ecological dynamics of plant and rodent populations and satellite sensor readings will be needed to determine which of these alternatives may apply (19,20).

Field validation of interpretations, which is critical to testing hypotheses derived from satellite data, should also be applied to the epidemiologic analyses of HPS risk. Our approach associates three TM bands and elevation with human risk. Interpretation of what these bands detect in the environment varies (soil moisture, soil type, and vegetation structure) (19). Our classification is being used to identify other sites with similar reflectance patterns in the same bands and characterize the structure and dynamics of rodent reservoir populations. Preliminary analyses show a good relationship between HPS risk predicted from satellite imagery and ***P. maniculatus*** population abundance ($r = 0.92$).

The case-control model using high-resolution spatial data from satellite imagery supports previous epidemiologic observations indicating that changes in rodent population densities and HPS risk could occur dramatically over relatively short distances (6). Although extensive areas of high and low risk were evident (Figure 3), substantial interdigitation of these zones at higher resolution created a mosaic of high- and low-risk areas. This suggests possibly widespread "environmental islands" of suitable reservoir habitat imbedded within less suitable habitat and may account for the apparently focal nature of HPS outbreaks and the near random distribution of cases relative to their nearest neighbors observed in this study (Table 1, $k = 1$).

The results also support epidemiologic investigations indicating that the only measurable risk factor around HPS sites during the 1993 epidemic was the abundance of ***P. maniculatus*** (6).

Satellite imagery analysis provides an efficient survey of large geographic regions for environmental indicators of disease risk affecting human populations and has the potential to make surveillance of disease risk for rare zoonotic and vectorborne diseases practical for public health applications (18,20-26). For many diseases, the basis for the supposition that remotely sensed data will be useful for anticipating disease risk is that pathogen transmission is facilitated by arthropods, whose survival and reproduction are influenced by variations in temperature and humidity. The effect of climatic variability, however, on directly transmissible zoonotic agents maintained in vertebrate, especially mammalian reservoirs, is less certain and has received little attention.

Additionally, although the reason to assume a relationship between climate variability and infectious disease outbreaks is clear (27), few studies have evaluated whether this presumed relationship actually exists. This study indicates that if these relationships do occur, they are modulated by a number of poorly understood ecologic and social conditions that will require substantial detailed studies of the pathways influencing disease risk.

### Acknowledgments

We thank Anne Grambsch for assistance in the early development and planning of this project and to the state and local Outbreak Response Teams, CDC, IHS, and environmental health specialists and biologists from health departments and universities in New Mexico, Arizona, and Colorado, who collected the epidemiologic data with such care.

Dr. Glass was supported in part by an Intergovernmental Personnel Agreement from the Centers for Disease Control and Prevention and NASA 97CAN01-0048. Drs. Patz and Shields were supported by the U.S. Environmental Protection Agency ORD STAR grant 96-NCERQA-1B and Dr. Patz received additional support from a cooperative agreement from the U.S. EPA Climate Policy and Assessment Division. Satellite imagery and supplies were provided through cooperative agreement CR823143 and grant 96-NCERQA-1B from EPA and cooperative agreement 97CAN01-0048 from NASA.

Dr. Glass is associate professor, W. H. Feinstone Department of Molecular Microbiology and Immunology, with a joint appointment in the Department of Epidemiology, The Johns Hopkins School of Public Health; he is director of the Spatial Information Systems Unit, Program on Health Effects of Global Environmental Change. His area of interest is the ecology of rodent-borne zoonotic diseases.

### References

1. Nichol ST, Spiropoulou CF, Morzunov S, Rollin PE, Ksiazek TG, Feldmann HS, et al. Genetic identification of a hantavirus associated with an outbreak of acute respiratory illness. Science 1993;262:615-8.
2. Childs JE, Ksiazek TG, Spriopoulou CF, Krebs JW, Morzunov S, Maupin GO, et al. Serologic and genetic identification of ***Peromyscus maniculatus*** as the primary rodent reservoir for a new hantavirus in the southwestern United States. J Infect Dis 1994;169:1271-80.
3. Glass GE. Hantaviruses. Current Opinion in Infectious Diseases 1997;10:362-6.
4. Engelthaler D, Mosley D, Bryan R, Cheek J, Levy C, Komatsu K, et al. Investigation of climatic and environmental patterns in hantavirus pulmonary syndrome cases in the Four Corners states. Emerg Infect Dis 1999;5:87-94.
5. Parmenter RR, Brunt JW, Moore DI, Ernest S. The hantavirus epidemic in the Southwest: rodent population dynamics and the implications for transmission of hantavirus-associated adult respiratory distress syndrome (HARDS) in the Four Corners region. 41:1-44. University of New Mexico: Sevilleta LTER Publication; 1993.
6. Childs JE, Krebs JW, Ksiazek TG, Maupin GO, Gage KL, Rollin PE, et al. A household-based, case-control study of environmental factors associated with hantavirus pulmonary syndrome in the southwestern United States. Am J Trop Med Hyg 1995;52:393-7.
7. Mills JN, Ksiazek TG, Peters CJ, Childs JE. Long-term studies of hantavirus reservoir populations in the southwestern United States: a synthesis. Emerg Infect Dis 1999;5:135-42.
8. Mills JN, Ksiazek TG, Ellis BA, Rollin PE, Nichol ST, Yates TL, et al. Patterns of association with host and habitat: antibody reactive with Sin Nombre virus in small mammals in the major biotic communities of the southwestern United States. Am J Trop Med Hyg 1997;56:273-84.
9. Anderson RM, May RM. Infectious diseases of humans: dynamics and control. Oxford: Oxford University Press; 1991.
10. Simonsen L, Dalton MJ, Breiman RF, Hennessy T, Umland ET, Sewell M, et al. Evaluation of the magnitude of the 1993 hantavirus outbreak in the southwestern United States. J Infect Dis 1995;172:729-33.
11. Eastman JR. IDRISI for Windows. Worcester (MA): Clark University; 1995.
12. Chavez PS. Atmospheric, solar, and MTF corrections for ERTS digital imagery. American Society of Photogrammetry and Remote Sensing, Proceedings. 1975.
13. Cuzick J, Edwards R. Spatial clustering for in homogeneous populations. Journal of the Royal Statistical Society, Series B 1990;52:73-104.
14. Collett D. Modelling binary data. 2nd ed. London: Chapman & Hall; 1994.

15. Fletcher RH, Fletcher SW, Wagner EH. Clinical epidemiology–the essentials. Baltimore: Williams & Wilkins; 1982.
16. Simes RJ. An improved Bonferroni procedure for multiple tests of significance. Biometrika 1986;73:751-4.
17. Glass GE, Schwartz BS, Morgan JM, Johnson DT, Noy PM, Israel E. Environmental risk factors for Lyme disease identified with geographic information systems. Am J Public Health 1995;85:944-8.
18. Linthicum KJ, Bailey CL, Davies FG, Tucker CJ. Detection of Rift Valley fever viral activity in Kenya by satellite remote sensing imagery. Science 1987;235:1656-9.
19. Sabins FF. Remote sensing principles and interpretation. New York: W.H. Freeman and Company; 1997.
20. Beck LR, Rodriguez MH, Dister SW, Rodriguez A, Washino D, Roberts RK, et al. Assessment of a remote sensing-based model for predicting malaria transmission risk in villages of Chiapas, Mexico. Am J Trop Med Hyg 1997;56:99-106.
21. Kitron U, Bouseman, JK, Jones CJ. Use of the ARC/INFO GIS to study the distribution of Lyme disease ticks in an Illinois county. Prevent Vet Med 1991;11:243-8.
22. Malone JB, Fehler DP, Loyacano AF, Zukowski SH. Use of LANDSAT MSS imagery and soil type in a geographic information system to assess site-specific risk of fascioliasis on Red River basin farms in Louisiana. Tropical Veterinary Medicine: Current Issues and Perspectives 1992;653:389-97.
23. Rogers DJ, Randolph SE. Distribution of tsetse and ticks in Africa: past, present and future. Parasitology Today 1993;9:266-71.
24. Kitron U, Otieno LH, Hungerford LL, Odulaja A, Brigham WU, Okello OO, et al. Spatial analysis of the distribution of tsetse flies in the Lambwe Valley, Kenya, using Landsat TM satellite imagery and GIS. Journal of Animal Ecology 1996;65:371-80.
25. Kitron U, Kazmierczak JJ. Spatial analysis of the distribution of Lyme disease in Wisconsin. Am J Epidemiol 1997;145:1-8.
26. Malone JB, Abdel-Rahman MS, El Bahy MM, Huh OK, Shafik M, Bavia M. Geographic information systems and the distribution of ***Schistosoma mansoni*** in the Nile delta. Parasitology Today 1997;13:112-9.
27. Patz JA, Epstein PR, Burke TA, Balbus JM. Global climate change and emerging infectious diseases. JAMA 1995;275:217-23.
